# Proctitis after stapled hemorrhoidopexy is an underestimated complication of a widely used surgical procedure: a retrospective observational cohort study in 129 patients

**DOI:** 10.1186/s13037-015-0081-6

**Published:** 2015-11-10

**Authors:** Peter C. Ambe, Dirk R. Wassenberg

**Affiliations:** Helios Klinikum Wuppertal, Department of Surgery II, University of Witten-Herdecke, Heusnerstr. 40, 42283 Wuppertal, Germany; Chirurgische Klinik, St. Remigius Krankenhaus Opladen, An St. Remigius 26, 51379 Leverkusen, Germany

**Keywords:** Hemorrhoids, Stapled hemorrhoidopexy, Procedure for prolapsed hemorrhoids, Hemorrhoidectomy, Proctitis

## Abstract

**Background:**

Hemorrhoidal disease is highly prevalent in the western world. Stapled hemorrhoidopexy also known as the procedure for prolapsed hemorrhoids (pph) has been shown to be superior to conventional hemorrhoidectomy with regard to postoperative pain, length of hospital stay and early return to work. Proctitis following stapled hemorrhoidopexy has not been reported previously. Herein, we report our experience with proctitis in patients following stapled hemorrhoidopexy and question if proctitis could be a complication of stapled hemorrhoidopexy.

**Materials and methods:**

A retrospective analysis of the data of patients undergoing stapled hemorrhoidopexy with the PPH03 in the coloproctology unit of the department of surgery of a primary care hospital in Germany within a 5-year period was performed. All cases were managed and followed up by a single attending surgeon with expertise in coloproctology.

**Results:**

129 patients were included for analysis including 21 cases with grade 2, 103 cases of grade 3 and 5 cases of grade 4 hemorrhoids. The median duration of surgery was 20 min. 17 complications including two recurrences were recorded. Post-pph proctitis was recorded in 14 cases (10.9 %). Post-pph proctitis was not associated with gender, extent of hemorrhoidal disease, BMI and ASA (*p* >0.05). All cases recovered within 4 weeks following management with nonsteroidal anti-inflammatory drugs and suppositories.

**Conclusion:**

Proctitis could be a complication of stapled hemorrhoidopexy with a good response to conservative treatment with suppositories.

## Introduction

Hemorrhoids are enlarged vascular cushions in the anal canal which become symptomatic secondary to engorgement or prolapse. Hemorrhoidal disease is highly prevalent in the western world with an estimated incidence of 4–30 % in the UK and USA [1–3]. Surgical management is indicated following the failure of nonoperative treatment options. Surgical resection can be achieved using conventional hemorrhoidectomy techniques [4–7] or via stapled hemorrhoidopexy also known as the procedure for prolapsed hemorrhoids (PPH) first described by Antonio Longo in 1998 [8]. The goal of PPH is the removal of abnormally enlarged hemorrhoidal tissues followed by a reduction of the remaining hemorrhoidal tissue into its normal anatomic site within the anal canal.

The birth of PPH however was associated with some controversy. Reported cases of devastating complications and pain following the procedure lowered its initial acceptance amongst surgeons [9–11]. The procedure however, was shown in a series of meta-analyses and systematic reviews not only to be safe and effective but also to be superior to conventional hemorrhoidectomy with regard to postoperative pain and early return to work [12, 13]. In 2007 PPH was recommended by the National Institute for Health and Care Excellence (NICE) as an option for the surgical management of patients with prolapsed internal hemorrhoids [14] and has become an established procedure. Proctitis has not been previously reported as a complication of PPH. Herein, we report our experience with post – pph proctitis and question if this could be a complication of PPH.

## Patients and methods

Following the approval of the hospital’s ethics committee, a search of the departmental database for patients undergoing surgery for hemorrhoidal disease from January 2009 to December 2013 was performed. The charts of these patients were retrospectively reviewed. Baseline data including sex, age, body mass index (BMI) and co-morbidity score as defined by the American Society of Anesthesiologists (ASA) were recorded for each patient.

Patient’s recruitment was mostly via referral by primary care physicians or following presentation in the emergency department with hemorroidal bleeding or prolapsed hemorrhoids. Per departmental standards, cases with hemorrhoidal bleeding and prolapse were generally admitted and closely monitored. Conventional hemorrhoidectomy was performed in cases with persistent bleeding and in cases with irreducible prolapse. In all other cases, surgery was performed about 12 weeks after conservative treatment.

All patients were seen in our outpatient proctology office by an attending surgeon with expertise in coloproctology. During the outpatient consultation, proctoscopy was performed in all cases and the degree of hemorrhoidal disease was documented. Colonoscopy was recommended and performed in selected cases prior to surgery.

Surgery was performed as inpatient procedure under general anesthesia. Patients were given an enema shortly before surgery. A single shot antibiotic was administered at the beginning of surgery in all cases. The intraoperative extent of hemorrhoidal disease was documented in the lithotomy position using the classification by Goligher [15]. All cases were managed by the same specialist using a PPH03 circular stapler (PPH Hemorrhoidal Circular Stapler, Ethicon) as described elsewhere [9]. Generally, the purse string was placed about 2 cm above the hemorrhoidal pendicle and approximately 3 cm oral to the dentate line.

Patients were discharged if the following criteria were met: postoperative defecation without relevant amount of blood in stool, no significant increase in postoperative c - reactive protein and/or white blood count and good pain control with pain killers per os. A stool softener was recommended in all cases postoperatively.

Follow-up was performed by the same specialist, usually 14 days after discharge in the outpatient proctology office and included a proctoscopic examination in all cases. Follow-up continued thereafter as needed.

The operative records were reviewed for information on disease severity and the course of surgery. The final discharge notes were consulted for information on the immediate postoperative course. Histopathology reports were reviewed in all cases. The outpatient charts were reviewed for data on follow – up and complications post discharge.

The data collected was analyzed using the Statistical Package for Social Science (SPSS®), IBM, version 22. Continuous variables were described using absolute case numbers and percentages while central tendencies were described with medians and interquartile ranges. Significances were calculated using the chi square test with levels of significance set at *p* < 0.05.

**Fig. 1:**
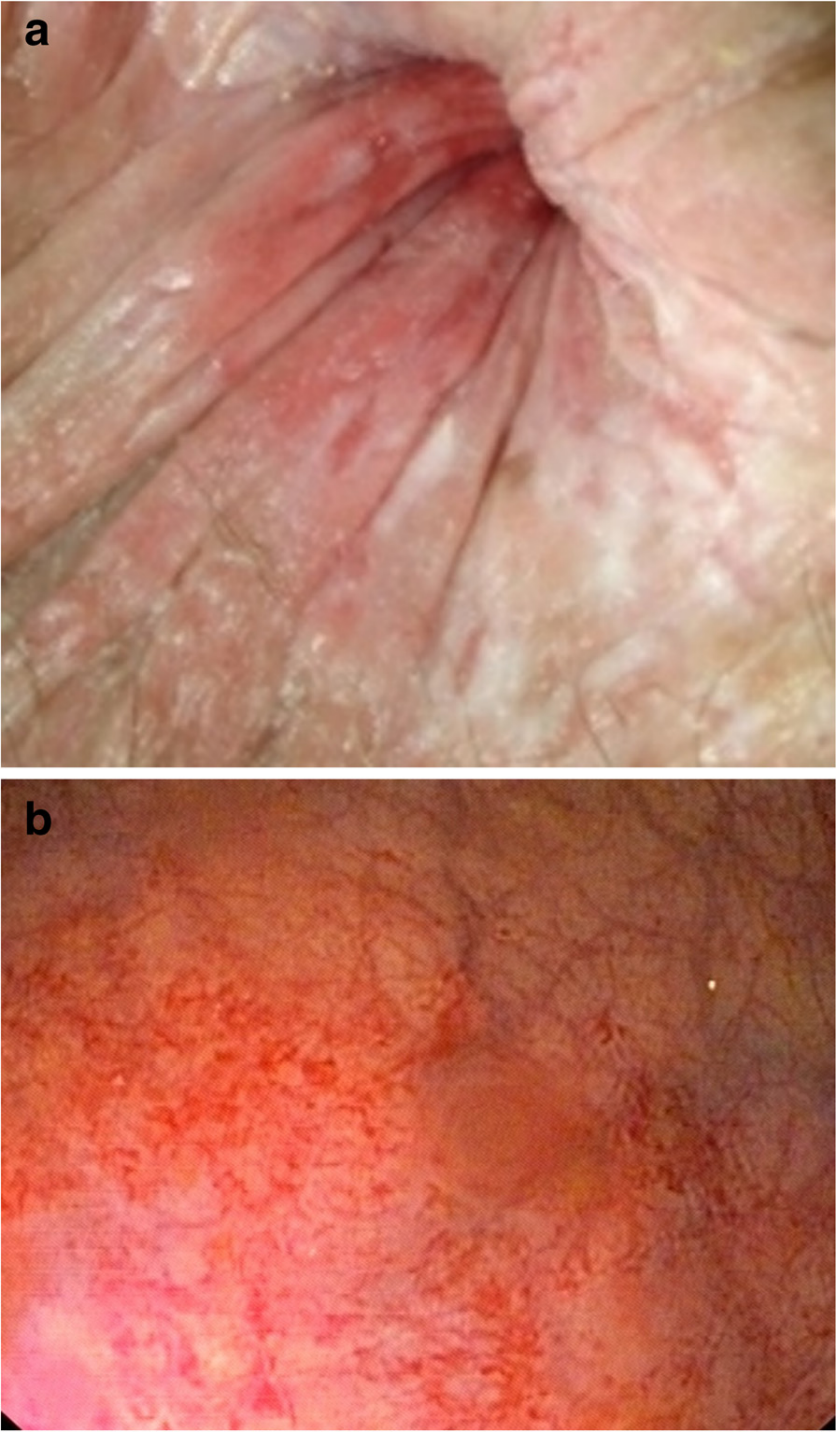
**a** Macroscopic appearance of the anal mucosa on physical examination. **b** Proctoscopic appearance of proctitis. The anal mucosa is erythematous, edematous and vulnerable
- after exclusion of other possible causes of proctitis e.g. inflammatory bowel disease, stool impaction, etc.

In this series, post-pph proctitis was defined as an inflammation of the anal mucosa after PPH. The diagnosis was suspected based on patients’complaints and the macroscopic appearance of the anal mucosa on proctoscopy:

- patient’s complaints: pruritus, blood or mucus in stool after discharge following an initially uneventful postoperative recovery,
- Macroscopic mucosa appearance during proctoscopy, i.e. erythema, edema and contact vulnerability (Fig. 1a and b) and

**Fig. 2:**
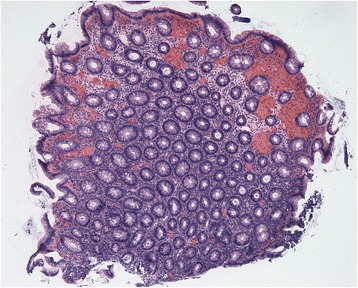
Histopathology. Biopsies of affected mucosa shallow cysts and features of epithelial regeneration following a subacute inflammation

The diagnosis was confirmed following the identification of epithelial regeneration with shallow crypts (Fig. 2) in mucosa biopsies of affected patients.

The primary outcome was the incidence of post-pph proctitis. Secondary outcomes included the rates of complication in general, reintervention, readmission and recurrence.

## Results

**Fig. 3:**
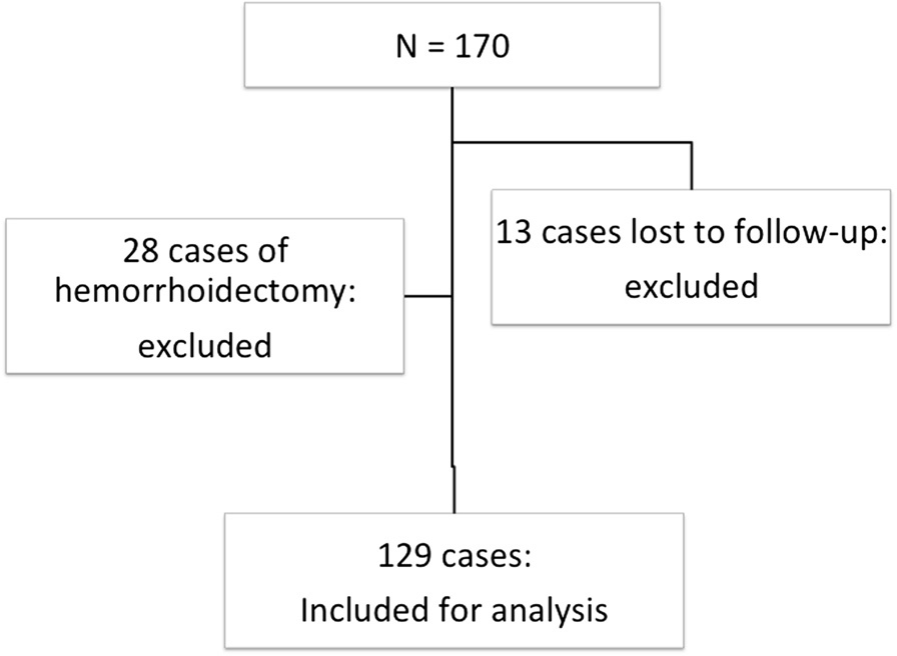
Distribution of the study population. 129 cases were included for analysis after excluding cases of hemorrhoidectomy and patients lost during follow-up

Within the period of investigation, 170 patients with symptomatic hemorrhoidal disease were managed in our department. Conventional hemorrhoidectomy was performed in 28 cases while 142 cases were managed with PPH. After excluding 13 cases without follow-up data, 129 cases were included for analysis, Fig. 3. Table 1 summarizes the demographic characteristics of the study population. Secondary procedures were done in 36 cases including 12 cases with anal fibroma and 24 cases with skin tags. Muscle fibers were identified in the resected hemorrhoidal tissues in 19 cases on histopathology.

**Table 1:** Summary of the baseline features of the study population

Demographic characteristics	
Features	Results
Gender (F/M)	68/61
Median Age	50 year
Interquartile range	19 years
ASA 1	42 (32.6 %)
2	69 (53.5 %)
3	18 (14.0 %)
BMI (kg/m^2^)	
≤ 25.0	57 (44.2 %)
25.1–30.0	49 (38.0 %)
30.1–35.0	16 (12.4 %)
> 35.0	7 (5.4 %)

*F* female, *M* male, B*MI* body mass index, *ASA* American Society of Anesthesiology Score

Over 79.8 % (103 cases) of the study population was managed for grade III hemorrhoids. Grade II hemorrhoids were present in 21 cases (16.3 %) while grade IV hemorrhoids were managed in five cases (3.9 %). The median duration of surgery was 20 min with an interquartile range of 10 min. The median length of postoperative hospital stay was 3d with an interquartile range of 1d.

Complications, both surgical and medical were recorded in 17 cases (13.2 %). Twelve (11 surgical and one medical) complications (9.3 %) including four cases of staple line dehiscence, two cases of recurrence, bleeding and stenosis, one case of abscess formation in the anal canal, and transient arrhythmia were registered in the group with grade III hemorrhoids. Five complications (3.9 %) including one case of dysparuenia, staple line stenosis, mucosa rupture, incontinence and pre-sacral abscess formation were recorded in the group with grade II hemorrhoids. This difference was not statistically significant, *p* = 0.18.

Complications were surgically managed in nine cases (6.9 %) including three patients with staple line dehiscence, two patients with postoperative bleeding from the staple line, postoperative abscess formation and recurrence (1.5 %). Both cases of bleeding occurred during hospital stay. Readmission was necessary in the remaining seven cases (5.4 %).

Routine postoperative proctoscopy two weeks after surgery revealed proctitis in 14 patients (10.9 %). 13 of these patients (10.1 %) presented with grade III hemorrhoids and one (0.8 %) presented with grade II hemorrhoids. This difference was not statistically significant, *p* = 0.41. Postoperative proctitis was independent of sex (*p* = 0.53), BMI (*p* = 0.63) and ASA score (*p* = 0.44).

There was no significant difference in the incidence of post-pph proctitis in patients with and without secondary procedure (*p* = 0.71). This was true for the presence or absence of muscle fibers in the resected hemorrhoidal specimens on histopathology (*p* = 0. 66).

Postop-pph proctitis in all 14 cases was associated with blood and mucus in stool. Eight patients reported mild soiling and pruritus. Mild discomfort, especially during defecation was reported in four cases. All 14 cases were managed conservatively with nonsteroidal anti-inflammatory drugs (NSAID) and malasazine suppositories. All 14 cases were followed up on a weekly basis.

Symptoms resolved in all cases following conservative management within 4 weeks. There was no mortality in this series.

## Discussion

Hemorrhoidal disease is highly prevalent in industrialized nations with symptoms ranging from mild discomfort to obstructed defecation [16]. Surgical management is the main stay of treatment after failure of nonoperative options. PPH is associated with a reduction of postoperative pain, shorter hospital stay and earlier return to work [17, 18]. However, the long term rate of recurrence has been reported to be higher following PPH [19, 20] while rarely seen serious complications have been attributed to surgical errors [21, 22]. Herein, we report our experience with proctitis after PPH.

One hundred and twenty-nine cases managed with PPH were included for analysis. A vast majority of the study population presented with grade III hemorrhoids. The median duration of surgery was 20 min while the median length of hospital stay was 3d. The length of stay in this study appears rather too long compared to the complexity of the procedure, especially since PPH can be performed as an out-patient procedure. This trend however, must be blamed on the local health insurance policy in Germany.

Complications were recorded in 13.2 %, reintervention was performed in 6.9 % including two cases of recurrence (1.5 %), while readmission was necessary in 5.4 %. These data are in accordance with current literature [23].

Post-pph proctitis was recorded in 10.9 % of cases. Post-pph proctitis was clinically diagnosed in patients with new onset of pruritus and blood or mucus in stool after excluding other clinically apparent causes of proctitis e.g. inflammatory bowel disease and fecal impaction.

Blood or mucus discharge was reported by all affected patients. Soiling and pruritus was reported in over 50 % of cases, while mild discomfort during defecation was reported in some cases. The mucosa of affected patients appeared erythematous, edematous and vulnerable on contact during proctoscopy. Histopathology showed signs of subacute inflammation with epithelial regeneration and shallow cysts. These findings are in accordance with endoscopic findings of inflamed anorectal mucosa [24].

Interestingly, neither abdominal examination nor blood test was pathologic in patients with post-pph proctitis. Thus the pathology seems to be limited to the anal canal mucosa. Equally, pain did not seem to be an issue in these patients. Therefore, post-pph proctitis should not be confused with pain following stapled hemorrhoidopexy.

The pathophysiology of proctitis in these patients is not clear. Proctitis is generally thought to originate from the morgagnian crypts [24]. Therefore, it is thinkable that irritation of the morgagnian crypts at the time of surgery may play a role in the development of post-pph proctitis. Assuming that such irritated crypts need some time before becoming symptomatic may explain the latency of symptom onset.

Objectively definable risk factors for the development of proctitis following PPH could not be identified in this study. Post-pph proctitis was independent of ASA, BMI and gender. Equally, post-pph proctitis was not identified more frequently in patients undergoing secondary procedures like removal of anal fibroma or skin tags. Also, the depth of bite could not be identified as a risk factor since there was no significant difference in the incidence of post-pph proctitis amongst patients with or without muscle fibers in the resected hemorroidal specimen.

However, 90 % of post-pph proctitis was diagnosed in patients managed for third degree hemorrhoids. Although this finding was not statistically significant, probably due to the small size of the study population, extensive hemorrhoidal disease (grade 3 and 4) might be a risk factor for post-pph proctitis.

The role of histopathology in the diagnosis of post-pph proctitis is questionable. Besides, taking biopsy of the inflamed and vulnerable mucosa could cause profuse bleeding and potentially serious infectious complications. Therefore, our recommendation is that the diagnosis of post - pph proctitis should be made clinically using the above mentioned criteria (patient’s complaints, macroscopic mucosa appearance on proctoscopy, and history of recent PPH) after excluding other possible causes.

Post-pph proctitis was managed medically with malasazine suppositories and NSAIDs. Other forms of suppositories [25, 26] and orally administered calcium dobesilate have been reported to be effective for similar conditions [27, 28]. Hospital admission was not needed. Equally, systemic antibiotics were not administered. All patients recovered within 4 weeks of treatment.

Although proctitis following anorectal surgery has not been previously described, its natural course seems to be benign with little or no symptoms. Besides, symptoms of post-pph proctitis could be attributed to the surgery per se. The immediate association of proctitis with PPH however makes it a possible postoperative complication.

The cases recorded in this series could only be diagnosed because of our strict departmental follow-up practice. Therefore, the importance of postoperative follow-up cannot be over emphasized.

This study is limited by its retrospective design and the small size of the study population. Therefore, proctitis as a complication following stapled hemorrhoidopexy warrants further investigation.

## Conclusion

Proctitis could be a complication of stapled hemorrhoidopexy. Symptoms may include pruritus, blood or mucus in stool. The mucosa of involved patients appears markedly erythematous, edematous and is vulnerable on contact. Treatment is usually medical with suppositories and NSAID in an outpatient setup and recovery is expected within 4 weeks.

## Consent

Written informed consent was obtained from each patient for the publication of this study and any accompanying images.
